# An overview of the peer review process in biomedical sciences

**DOI:** 10.1177/10398562241231460

**Published:** 2024-02-08

**Authors:** Edward Miller, Michael James Weightman, Ashna Basu, Andrew Amos, Vlasios Brakoulias

**Affiliations:** Division of Psychological Medicine, 1415 The University of Auckland, Auckland, New Zealand; School of Medicine, 1066 The University of Adelaide, Adelaide, SA, Australia; College of Medicine and Public Health, Flinders University, Bedford Park, SA, Australia; 6804 Prince of Wales Hospital, Sydney, NSW Australia; Discipline of Psychiatry and Mental Health, UNSW, Sydney, NSW, Australia; Committee for Research, Royal Australian and New Zealand College of Psychiatry, Melbourne, VIC, Australia; School of Medicine and Dentistry, 104397 James Cook University, Townsville, QLD, Australia; School of Medicine and Translational Health Research Institute (THRI), Western Sydney University, Sydney, NSW, Australia; Specialty of Psychiatry, Faculty of Medicine and Health, The Univesity of Sydney, Sydney, NSW, Australia

**Keywords:** medical education, peer review, biomedical publishing, psychiatry

## Abstract

**Objective:**

This paper aims to provide an introductory resource for beginner peer reviewers in psychiatry and the broader biomedical science field. It will provide a concise overview of the peer review process, alongside some reviewing tips and tricks.

**Conclusion:**

The peer review process is a fundamental aspect of biomedical science publishing. The model of peer review offered varies between journals and usually relies on a pool of volunteers with differing levels of expertise and scope. The aim of peer review is to collaboratively leverage reviewers’ collective knowledge with the objective of increasing the quality and merit of published works. The limitations, methodology and need for transparency in the peer review process are often poorly understood. Although imperfect, the peer review process provides some degree of scientific rigour by emphasising the need for an ethical, comprehensive and systematic approach to reviewing articles. Contributions from junior reviewers can add significant value to manuscripts.

Academic peer review refers to the processes by which submissions to scientific journals are examined and altered, mostly prior to publication.^1^ These processes have evolved over time, and while some may be grounded in tradition, they are increasingly subject to scientific rigour. The first organised peer review process seems to have started in the United Kingdom in the early 18^th^ century, when the Royal Societies of Edinburgh and London began asking their members to evaluate submitted manuscripts. Articles were selected for publication based on their ‘novelty, ingenuity or importance’, and the Societies issued disclaimers explaining that they took no responsibility for the ‘truth of facts, soundness of reasoning, or accuracy of the calculations’ found within.^2^ As these original institutions often received more submissions than they could assess internally, this process provided a timely and straightforward method to determine which submissions should be published.^1^

Over time, this review method evolved in an indiscriminate way, with major variations between journals that were often susceptible to bias.^3^ Following the Second World War, the process gradually became more standardised and institutionalised, mirroring the evolution of medicine itself.^4^ The peer review process has been subject to increasing levels of scientific rigour including randomised controlled trials (RCTs) of the process itself, and greater consideration of ethical factors such as bias.^5^ The most effective method of peer review remains enigmatic and, despite advancements, its necessity, quality and validity have been questioned.^6–8^ Participating in peer review may therefore be an intimidating prospect for junior researchers or clinicians, who can lack a clear entry point into this field.^9^ This article will subsequently aim to demystify peer review by giving a concise overview of the subject. It will highlight some advantages and limitations of peer review, alongside proposed improvements and efforts to control the quality of published articles. Finally, it will reflect on approaches to peer review which may be useful for beginning reviewers.

## Types of peer review

The peer review process used by biomedical journals can be grouped into two general groups. The first method is a select-in approach, which is most often used by high-impact journals that pick a small group of submissions with the greatest relevance to their readership, and which are most likely to affect clinical practice. They may reject close to 90% of submissions, but the review process is usually thorough and time-consuming.^10^ The second group of journals takes a screening-out approach, whereby submissions can be accepted so long as they are within the scope of the journal and meet adequate ethical and methodological standards. These journals often have quicker turnaround and may be targeted by organisations who publish frequent routine or repeat studies.

The type of peer review offered will also differ depending on the type of submission preferred by the journal. For example, in addition to empirical research papers and systematic reviews, submissions may include opinion pieces, editorials, letters/correspondence, book/media reviews, bereavements, and sometimes more creative works such as poems, stories and personal reflections.^11^ These may not be distributed to the standard pool of peer reviewers, but could be screened, edited and reviewed by the chief editor or other specialty or deputy editors.

## The peer review process

Three general peer review systems operate in biomedical journals.^10^ In the simplest model, each submitted manuscript is managed by a single editor – either an editor-in-chief or a deputy or specialty editor. Editors scan for excessive spelling/grammar mistakes and confirm that submissions match the journal’s scope and address issues of significance to the journal’s readership.^11^ Individual editors will either reject a submission or select it for review and send it to multiple reviewers. This process is traditionally blinded, which means reviewers are usually not told the identity of submitting authors, although some studies suggest that reviewers can often identify who the authors are based on the nature of the submitted work.^12^ Authors will also usually not know the identity of the reviewers. Reviewers assess manuscripts against standardised criteria and provide detailed feedback aimed at eliminating errors and improving qualities such as the clarity, concision, and accuracy of the data reported and conclusions reached.

Individual journals have specific criteria for reviews, but a general outline is provided in Table 1. While peer review necessarily involves a degree of intersubjectivity and intuition informed by reviewers’ opinions and personal experience, the process should aim to be as objective as possible.^13^ Feedback should typically justify the reasoning behind suggested changes, including referencing other papers, and signposting areas of uncertainty, subjectivity or opinion within the response. Due to the time required to accurately appraise in this manner, this process may take several months in some cases.

**Table 1.** General outline of review criteria (adapted from Moher and Jadad)^16^

Domain	Considerations for reviewers
Importance of the research question	o Do authors contextualise and frame their research within a pre-established and valid research context? o Have authors addressed issues such as cost benefit analysis and other ethical justifications?
Originality of the work	o Have reviewers conducted a brief systematic review of other literature to confirm originality? o Have the authors conducted and included a review themselves?
Strengths and weaknesses (e.g. methodological, ethical)	o Can the methodology be appraised using an established standardised criteria, such as the CONSORT guidelines for reporting RCTs?
Presentation and clarity	o Do spelling, grammar and formatting mistakes affect the interpretation of the data and the argument?
Interpretation of results	o Have the authors justified the way in which results are interpreted and reported, including by addressing biases?
Limitations	o Have the authors addressed limitations of the study and how this may affect the results or their interpretation?
Suitability for publication	o Does the submission require minor/major changes, resubmission or rejection, and on what basis, with justification and citing evidence where possible?

Reviewers usually come from a pool of professionals with a high degree of knowledge about their field who volunteer their time. Many journals require reviewers to hold a PhD or equivalent qualification. Some reviewers declare that they have special expertise in specific areas of knowledge. If the pool contains no available volunteers with the required expertise for a particular article, it may be necessary to invite reviewers with less expertise in the topic of the paper. In some journals, it may be useful to include one reviewer with less expertise in the specific topic of the paper to ensure that the information provided in the paper can be understood by the average reader of the journal. While some articles benefit from the review of highly specialised experts, many will require only the attention of an interested professional with general knowledge about the domain, familiarity with the standard organisation of a scientific article and a reasonable facility with scientific writing. Junior doctors can provide a specific type of expertise in reviewing articles about the experience of training. Reviewers are often recruited over time through invitation by the editor or other reviewers, either through word of mouth or identified through their relevant publications, past submissions or even internet searches.^10^

The second general type of peer review uses an editorial board to review all submissions. This method selects from the same set group of board members to conduct all reviews and rarely outsources reviews to external reviewers. Reviews in this system typically take a long time, have less detail and requires board members with a high level of expertise. *Circulation* is a journal which has used this process.^14^

The third type uses a professional editorial team who are employed on a full-time basis to review articles. This system can still utilise external reviewers and may act as a hybrid between the first and second types of peer review. This method is commonly used by large and well-resourced journals, such as *The Lancet*. Other journals, such as the *British Medical Journal* (BMJ), still employ this method but require final review by their editorial board, who have the last say before publication.^15^

In all three models, editors may resolve disagreements between reviewers and authors by soliciting additional reviews. Some journals formally specify how disputes will be mediated, whilst other reviewers may also become involved if the paper is of a high technical standard. Papers containing statistics may often undergo a completely separate review by a statistician, or the journal’s nominated statistical editor, prior to publication.^10^

## Advantages and limitations of peer review

The peer review process is universally acknowledged to be imperfect, but there is no better alternative available at present.^17^ It is often criticised as slow, expensive, time-consuming, highly subjective, and prone to bias, error and fraud.^17^ Peer review is also far from certain to produce material that meets adequate levels of reliability and relevance,^18^ and is over-represented by reviewers from high-income countries, who may be more likely to accommodate a lack of financial reimbursement than reviewers from emerging regions.^19^ Authors have argued for the process to be more openly accessible and transparent.^20^ Nevertheless, some evidence suggests that peer review does improve the overall quality of published work. A study published in the *Lancet* demonstrated that peer reviewing and editing submitted manuscripts significantly improved their quality when measured on a blinded rating scale by 400 readers of the journal who had varying degrees of specialisation.^21^ Another descriptive analysis of submitted manuscripts to the journal *Annals of Internal Medicine* identified that changes made during the peer review process were most likely in response to the manuscript having either too much or too little information, inaccurate or misplaced information, and structural problems.^22^

Different types of peer review also have different strengths and weaknesses. Blinding facilitates honest, critical reviews, but it may make idea theft more likely as submitting authors cannot know who is reading their work.^23^ Open reviews, where reviewers and authors know each other’s identities, including publishing the reviewer’s information alongside the paper, help to acknowledge the reviewer’s time and work, and may reduce bias.^23^ Transparent reviews take this one step further and aim to publish the full transcript of the peer review process. This is intended to increase accountability and opportunity for the reader to critique the process, but it may be harder to find reviewers to participate in this process.^23^ An RCT by the *British Journal of Psychiatry* (BJP) found that compared to anonymised reviews, transparent reviews were higher quality, more courteous, took longer to complete, and that named reviewers were more likely to recommend publication.^24^ *BJP* now operates single-blind review, whereas journals such as *BMJ* use fully open review.^25^

Novel methods to address the limitations of traditional peer review include collaborative review, where reviewers collaborate on a single report; preprints, where papers are submitted to an online repository before formal review, allowing early access and opportunity for academic scrutiny; and post-publication review, where experts survey paper after it has been published. These methods have a different set of limitations to quality and transparency than traditional peer review.^23^

## Tips and tricks for new reviewers

Taking these considerations into account, this section provides some suggestions for new reviewers. The golden rule is to be courteous, constructive and to ‘do as you would be done unto’.^15^ Read the article a few times, note surface-level reflections before attempting a detailed critique, and re-visit your response over several days to refine what you have written before submitting your review. A short summary at the start of your review helps reassure authors that you have understood their paper and have a good understanding of their argument, which may make it more likely that they will follow your suggestions. Follow your journal’s review instructions when providing your report, separating responses into ‘general’ and ‘specific’ domains to make it easier for the authors to digest. Setting out comments in line with the sections of the manuscript and labelling them by page, paragraph and line is very helpful for editors and authors. Other tips and tricks for new reviewers are summarised in Table 2.

**Table 2.** Other tips and tricks for new reviewers (adapted from Moher and Jadad)^16^

Tip	Reason
Accept an invitation to peer review only if you have the knowledge and time to do so	o Check for abstract and cover letter o Ask whether you are familiar with the content o Consider conflicts of interest
Protect time	o Expect to take up to 8–12 h per review if you are new o Confirm review deadline and set reminders
Follow a systematic process	o Try to be as objective and systematic as possible o Recognise individual limitations and acknowledge subjective judgements
Ask for help	o Do not be afraid to ask for advice when reviewing manuscripts o Ask for senior colleagues to provide feedback on your reviews o Engage with online resources and training o Practice critiquing articles in a journal club format
Be clear and constructive	o Provide feedback which is open and honest when critical but still respectful and constructive
Have confidence	o Research shows that junior reviewers often provide the highest quality work^23,24^

## Conclusions

The academic peer review process of the biomedical science field has evolved rather haphazardly over time, but is now increasingly subject to scrutiny and the demand for higher standards. This paper has provided an overview of the processes, including the types of peer review and their advantages, limitations and areas for improvement. Peer review can be conducted in a blinded or an unblinded manner, and peer reviewers can be guided by a comprehensive and systematic approach to completing reviews. Peer reviewers are recruited from a pool of professionals to provide a balance of content knowledge, methodological expertise and diverse viewpoints in which junior colleagues have a valuable part to play.
